# ASO Author Reflections: A Simpler Predictor of Postoperative Delirium in Elderly Gastric Cancer Patients

**DOI:** 10.1245/s10434-024-16094-y

**Published:** 2024-09-13

**Authors:** Kazuyoshi Yamamoto

**Affiliations:** Department of Gastroenterological Surgery, Osaka University Graduate School of Medicine, Osaka, Japan

## Past

Patients undergoing surgery are getting older. Therefore, comprehensive geriatric assessment (CGA) to assess preoperative risk has become popular in recent years.^1,2^ However, not all hospitals have geriatricians, and CGA is time-consuming, so it is not available for all elderly patients.

## Present

We retrospectively studied CGA and other clinical factors in postoperative gastric cancer patients aged 75 years and older from 2010 to 2020 to explore more convenient methods of predicting postoperative delirium. The result, postoperative delirium might be more easily predicted based on the combination of MMSE and ECOG-PS in elderly patients with gastric cancer undergoing gastrectomy.^3^

## Future

As the world becomes an increasingly elderly society, there is a need to explore more comprehensive and simplified methods of risk assessment for the elderly.
